# Identifying facilitators of and barriers to the adoption of dynamic consent in digital health ecosystems: a scoping review

**DOI:** 10.1186/s12910-023-00988-9

**Published:** 2023-12-01

**Authors:** Ah Ra Lee, Dongjun Koo, Il Kon Kim, Eunjoo Lee, Hyun Ho Kim, Sooyoung Yoo, Jeong-Hyun Kim, Eun Kyung Choi, Ho-Young Lee

**Affiliations:** 1 Office of eHealth Research and Business, Seoul National University Bundang Hospital, Seongnam, Republic of Korea; 2 Interdisciplinary Program in Bioengineering, Seoul National University, Seoul, Republic of Korea; 3 School of Computer Science & Engineering, College of IT Engineering, Kyungpook National University, Daegu, Republic of Korea; 4 College of Nursing, Research Institute of Nursing Science, Kyungpook National University, Daegu, Republic of Korea; 5 Department of Pediatrics, Research Institute of Clinical Medicine, Jeonbuk National University, Jeonju, Republic of Korea; 6 Biomedical Research Institute of Clinical Medicine, Jeonbuk National University Hospital, Jeonju, Republic of Korea; 7 Department of Psychiatry, Seoul National University Bundang Hospital, Seongnam, Republic of Korea; 8 Department of Public Health Medical Services, Seoul National University Bundang Hospital, Seongnam, Republic of Korea; 9 Institute of Human Behavioral Medicine, Seoul National University Medical Research Center, Seoul, Republic of Korea; 10 Department of Medical Humanities and Medical Education, Kyungpook National University School of Medicine, Daegu, Republic of Korea; 11 Department of Nuclear Medicine, Seoul National University Bundang Hospital, Seongnam, Republic of Korea

**Keywords:** Dynamic consent, Digital health, Data sovereignty, Blockchain, Consent systems

## Abstract

**Background:**

Conventional consent practices face ethical challenges in continuously evolving digital health environments due to their static, one-time nature. Dynamic consent offers a promising solution, providing adaptability and flexibility to address these ethical concerns. However, due to the immaturity of the concept and accompanying technology, dynamic consent has not yet been widely used in practice. This study aims to identify the facilitators of and barriers to adopting dynamic consent in real-world scenarios.

**Methods:**

This scoping review, conducted in December 2022, adhered to the PRISMA Extension for Scoping Reviews guidelines, focusing on dynamic consent within the health domain. A comprehensive search across Web of Science, PubMed, and Scopus yielded 22 selected articles based on predefined inclusion and exclusion criteria.

**Results:**

The facilitators for the adoption of dynamic consent in digital health ecosystems were the provision of multiple consent modalities, personalized alternatives, continuous communication, and the dissemination of up-to-date information. Nevertheless, several barriers, such as consent fatigue, the digital divide, complexities in system implementation, and privacy and security concerns, needed to be addressed. This study also investigated current technological advancements and suggested considerations for further research aimed at resolving the remaining challenges surrounding dynamic consent.

**Conclusions:**

Dynamic consent emerges as an ethically advantageous method for digital health ecosystems, driven by its adaptability and support for continuous, two-way communication between data subjects and consumers. Ethical implementation in real-world settings requires the development of a robust technical framework capable of accommodating the diverse needs of stakeholders, thereby ensuring ethical integrity and data privacy in the evolving digital health landscape.

**Supplementary Information:**

The online version contains supplementary material available at 10.1186/s12910-023-00988-9.

## Background

Digital health integrates information and communication technology with healthcare [1, 2]. This convergence enables 4P medicine-prediction, prevention, personalization, and participation-to predict life-threatening diseases, prescribe personalized treatments, improve healthcare quality, and reduce medical costs [3–5]. Digital health relies on personal health data, including clinical, genomic, and exogenous information [6–8]. Competition among major companies in health data is fierce, with investments in digital health care startups rising annually [9, 10]. This shows the considerable interest in health data. Nevertheless, in light of these technological advancements, there is an ethical obligation for judicious data management to respect the rights, privacy, and autonomy of individuals. Most importantly, there is a growing recognition that digital research ethics require more appropriate consent methods.

Several ethics guidelines and principles underpin consent in digital health settings [11]. Following World War II, the Nuremberg Code established ethical standards in medical research by emphasizing voluntary, informed, and legally valid behavior [12]. According to the Declaration of Helsinki, informed consent, which includes a thorough explanation of the research’s purpose, risks, and benefits, should be provided to participants [13]. Likewise, the Belmont Report highlighted informed consent based on respect for individuals, including information, voluntariness, and comprehension [14]. These foundational ethical documents influence digital health research and practice by providing historical contexts and informing contemporary ethical guidelines.

Beyond these practical issues, there exist deeper ethical dilemmas. Not truly informed consent undermines ethical research and application, risking individual rights violations and eroding trust in digital health innovations [15]. In certain jurisdictions, consent may function as a legal authorization permitting consumers to access personal health data, though its universal applicability is contingent upon specific regional and contextual factors [16]. Consent allows data subjects to exercise self-determination and maintain sovereignty over their health data. Currently, specific and broad informed consent are the most common strategies for obtaining consent [17, 18]. Table 1 summarizes commonly used consent practices and their ethical implications.

**Table 1:** An overview of conventional consent practices

Features	Specific informed consent	Broad informed consent
Pros	$\bullet$ respect for individual rights	$\bullet$ efficiency in consent procedures
	$\bullet$ informed decision-making	$\bullet$ flexibility for future data usage
	$\bullet$ accountability & liability	$\bullet$ allowing broader use of data
Cons	$\bullet$ time-consuming & costly	$\bullet$ lack of autonomy
	$\bullet$ consent fatigue	$\bullet$ consent with limited information
	$\bullet$ lack of feasibility	$\bullet$ difficulties with withdrawal
		$\bullet$ not allowing delivery of afterward information
Ethical problem	$\bullet$ barriers to participation	$\bullet$ potential for misuse of data
	$\bullet$ additional burden of research	$\bullet$ lack of transparency
	$\bullet$ incomplete or biased information	$\bullet$ insufficient willingness

Specific informed consent is a well-established method that guarantees voluntary participation in medical research [19]. It provides research details and asks individuals about their willingness to participate. Participants should understand the study’s potential risks and benefits and have the right to withdraw consent at any time. Nevertheless, specific informed consent imposes practical challenges in digital health ecosystems [20]. When providing data, the research value may not be fully known, making specific informed consent difficult to obtain [21]. It is also an ethical and administrative burden to ask donors for consent each time new research is conducted. Re-consenting is time-consuming and burdensome. In long-term projects, such as cohort studies, initial information may become outdated or insufficient [22]. Generally, the specific informed consent fails to accommodate these evolving participant preferences.

The 2017 US Common Rule revision introduced broad informed consent, going beyond specific informed consent [23, 24]. Broad informed consent is an alternative to obtaining consent for unspecified future data usage [25, 26]. Broad consent, unlike specific consent, streamlines the consent procedure by not requiring a specific range for each use. Institutions and researchers with broad consent commit to ethical governance structures to protect participant privacy and rights when using collected data. However, this model is ethically problematic because participants may not be fully informed of research activities they consent to, potentially undermining informed consent [23, 27, 28]. As research evolves, the broad consent may not reflect participants’ preferences, leading to abusive data use [29]. Furthermore, if there are significant findings regarding the health of donors, using non-identifiable data for broad consent may impose ethical concerns. The 2016 Taipei Declaration by the World Medical Association restricts the range of broad informed consent [30]. The Council for International Organizations of Medical Sciences also argued that proper data governance is necessary for broad consent [31].

Dynamic consent (DC) emerges as a beacon of an ethical solution. DC provides individuals with granular control and continuity, promoting trust, transparency, and autonomy in digital health [32]. It enhances trust between individuals and researchers by continuously providing up-to-date information for informed decision-making [33, 34]. DC is more interactive and user-friendly than traditional consent practices as it is tailored to individual preferences [35]. Given the dynamic nature of digital health research, with its constantly evolving data and methodologies, conventional consent models may become inadequate and burdensome. Despite its benefits, DC has not been widely adopted due to conceptual and technological challenges [36]. A scoping review is needed for an in-depth understanding of domain-specific knowledge by presenting an overview of the literature as a multifaceted and focused summary [37]. Additionally, it can provide potential future research directions by identifying research gaps [38]. No scoping review of DC has been conducted, to our knowledge.

Therefore, this study conducts a scoping review on DC to answer the following research questions: RQ1. What are the current research trends in DC? RQ2. What are the characteristics that contribute to the flexible and ever-changing nature of DC? RQ3. What are the challenging aspects of introducing DC in the health domain? This study aims to identify the facilitators of and barriers to the adoption of DC in digital health environments. By analyzing the previous literature, it is possible to gain insights on the potential consequences and considerations for the implementation of DC systems in real-world settings. This study contributes to the existing body of knowledge concerning the adoption of DC. The findings from this study would promote an ethical digital health ecosystem that respects individuals’ rights and interests.

## Methods

### Review protocol

**Fig. 1:**
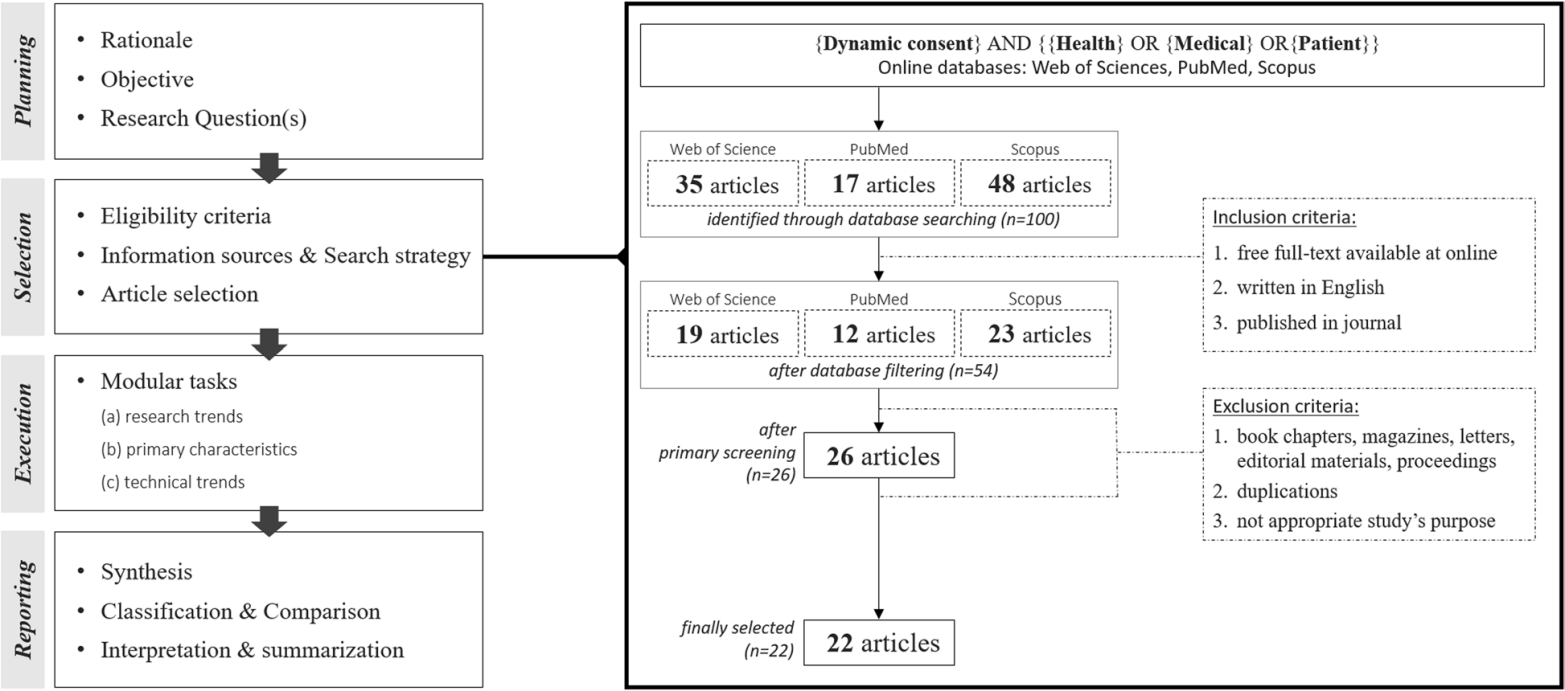
An overview of the review protocol & the article selection process

Figure 1 illustrates an overview of the review protocol used in this study. This review was conducted in accordance with the PRISMA Extension for Scoping Reviews (PRISMA-ScR) guidelines [39]. The protocol consists of four stages: planning, selection, execution, and reporting. The execution phase includes modular tasks: (a) examining research trends, including academic contributions; (b) defining the current state of knowledge through a synopsis of principal findings; and (c) obtaining implications from realistic perspectives, especially regarding technological advancement and implementation. The modular tasks enable a concentrated synthesis and analysis of selected articles, as well as the acquisition of pertinent insights to address the research questions.

### Eligibility criteria

Articles were selected based on inclusion and exclusion criteria. This study included articles that are freely accessible in digital databases, written in the English language, and published in journals or conference proceedings. Only peer-reviewed literature was selected to provide a stronger foundation and evidence-based information through a synthesis of previous studies. Book chapters, magazines, letters, editorial materials, and conference proceedings were not included. Duplicates or those unsuitable for research purposes were also excluded.

### Information sources & search strategy

This study uses three databases: Web of Sciences, PubMed, and Scopus, all of which have been identified as popular sources of information in the health domain. Following a database search in December 2022, four keywords-“Dynamic consent,”, “Health,” “Medical,” and “Patient”—were selected. The search syntax was set by combining the four selected keywords as follows: *Dynamic consent* AND *Health* OR *Medical* OR *Patient*.

### Article selection

The article selection process is presented in Fig. 1. An initial search was conducted in three online databases using the search syntax comprising the selected keywords. A total of 100 articles were discovered on Web of Science, PubMed, and Scopus, which yielded 35, 17, and 48 results, respectively. After filtering articles based on the selection criteria, 46 articles were removed, and 54 articles remained. 37 were not freely available in full text online, 2 were not written in English, and 7 had not been published in a journal. The articles were then extracted from each database and integrated. Twenty-eight articles were removed because of duplication. Finally, two authors read the remaining article abstracts and removed four that were inappropriate for this study.

### Data synthesis

This study conducts a thematic analysis to classify key concepts from the selected articles [40]. This thematic analysis aims to provide a comprehensive overview of the subject’s historical trajectory, user perspectives, and technical dimensions to illuminate valuable insights into the breadth and development of this area. After thorough review of the selected articles, a thematic framework based on the most significant outcomes was defined. One researcher initially assigned each study a principal theme within this framework, and a second researcher validated them. Discrepancies were discussed with other authors and resolved.

## Results

### Research trends

#### Overview

This section outlines one of the principal outcomes. The profiling results to investigate current research trends on DC are displayed in Table 2. It comprises academic contributions, research themes, keywords, and application areas.

**Table 2:** An overview of selected articles

No	Authors	Year	Journal	Publisher	Country	Themes	Application areas
1 [41]	Kristin Solum Steinsbekk, Bjørn Káre Myskja & Berge Solberg	2013	European Journal of Human Genetics	Springer	Norway	Conceptual evolution	biobank
2 [32]	Jane Kaye et al.	2015	European Journal of Human Genetics	Springer	UK	Conceptual evolution	biobank
3 [42]	Daniel B. Thiel et al.	2015	Public Health Genomics	Karger	USA	Feasibility analysis	biobank
4 [43]	Hawys Williams et al.	2015	JMIR Medical Informatics	JMIR	UK	Conceptual evolution	medical research
5 [44]	Harriet J.A. Teare et al.	2015	Digital Health	SAGE	UK	Feasibility analysis	biobank
6 [45]	Karen Spencer et al.	2016	Journal of Medical Internet Research	JMIR	UK	Feasibility analysis	research
7 [35]	Isabelle Budin-Ljøsne et al.	2017	BMC Medical Ethics	BMC	Norway	Feasibility analysis	biobank, clinical trials, clinical research
8 [46]	Megan Prictor, Harriet J.A. Teare, and Jane Kaye	2018	Frontiers in Public Health	Frontiers	Australia	Conceptual evolution	biobank
9 [47]	Mohammad Firdaus Abdul Aziz and Aimi Nadia Mohd Yusof	2019	Asian Bioethics Review	Springer	Malaysia	Conceptual evolution	biobank
10 [48]	Nicholas Mamo et al.	2020	European Journal of Human Genetics	Springer	Malta	Technological advancement	biobank
11 [49]	Giuseppe Albanese et al.	2020	Journal of Ambient Intelligence and Humanized Computing	Springer	Switzerland	Technological advancement	clinical trials
12 [34]	George Despotou et al.	2020	Digital Health	SAGE	UK	Feasibility analysis	general practitioner
13 [50]	Joel E. Pacyna et al.	2020	European Journal of Human Genetics	Springer	USA	Feasibility analysis	biobank
14 [36]	Harriet J. A. Teare, Megan Prictor, and Jane Kaye	2020	European Journal of Human Genetics	Springer	UK	Conceptual evolution	biomedical research
15 [51]	Megan Prictor et al.	2020	Journal of Law, Medicine & Ethics	Cambridge University Press	Australia	Conceptual evolution	genomic data
16 [52]	Matilda A. Haas et al.	2021	European Journal of Human Genetics	Springer	Australia	Technological advancement	genomic research
17 [53]	Tong Min Kim et al.	2021	Applied Sciences	MDPI	South Korea	Technological advancement	medical data
18 [54]	Susan E. Wallace & José Miola	2021	BMC Medical Ethics	BMC	UK	Feasibility analysis	longitudinal cohort study
19 [55]	Faisal Albalwy, Andrew Brass, and Angela Davies	2021	JMIR Medical Informatics	JMIR	UK	Technological advancement	genomic data
20 [56]	Arno Appenzeller et al.	2022	Technologies	MDPI	Germany	Technological advancement	medical data, research
21 [57]	Ki Young Huh et al.	2022	Frontiers in Medicine	Frontiers	Sourth Korea	Technological advancement	clinical trials
22 [58]	Deborah Mascalzoni et al.	2022	European Journal of Human Genetics	Springer	Italy	Feasibility analysis	longitudinal study

#### Publications & citation counts

**Fig. 2:**
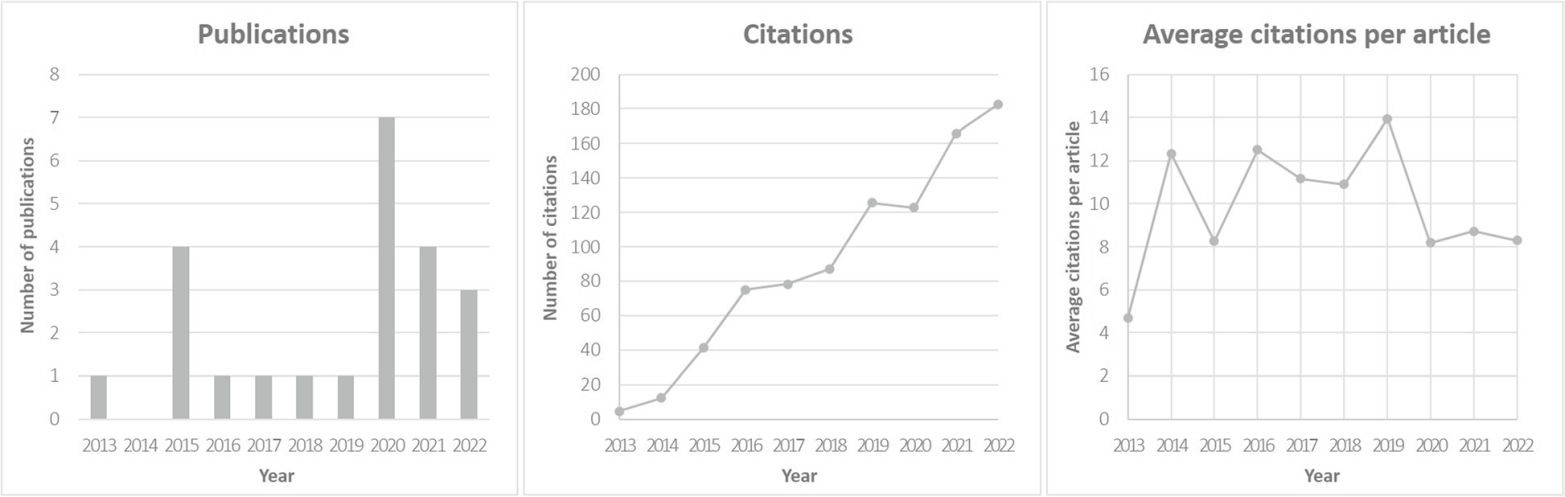
An overview of annual publications & citation counts

Figure 2 summarizes annual publications and citation counts. The average citation counts were calculated from the three databases used in the article selection process. The earliest publication year was 2013 [41]; and the most cited article was published in 2014 [32]. The most-cited article received approximately 188.33 citations over the past five years. Although there were few publications before 2019, citations had been rising steadily. From 2013 to 2016, only six articles were published, but they achieved 75 citations. Citations increased significantly in 2016, 2019, and 2021. The average number of citations per article also increased in 2016 and 2019, but not greatly in 2021. Even in years with high citation counts, publications only increased slightly. This suggests that the increase in citations was due to growing interest in DC, not just the number of publications.

#### Academic contributions

From the standpoint of the sources of the selected articles, ethics and genetics were the most prevalent areas of published journals. The close relationship between DC and ethics stems from its emphasis on the data sovereignty of individuals. DC is also intricately connected to genetics because its principal application area is biobanks. Besides these areas, the journals also covered digital health and medical information. DC operates based on digital interfaces as opposed to conventional consent mechanisms; therefore, it is inextricably linked to the field of information and communication technology.

Scholarly contributions to DC have originated from a diverse range of sources, transcending any particular journal or publisher. The majority of articles appeared in the European Journal of Human Genetics ($n = 7$), published by Springer. The other sources were Digital Health ($n = 2$) from SAGE; The Journal of Law, Medicine & Ethics ($n = 1$) from Cambridge University Press; BMC Medical Ethics ($n = 2$) from BMC; the Journal of Medical Internet Research ($n = 1$) and JMIR Medical Informatics ($n = 2$) from JMIR Publications; the Journal of Ambient Intelligence and Humanized Computing ($n = 1$) and Asian Bioethics Review ($n = 1$) from Springer; Frontiers in Medicine ($n = 1$) and Frontiers in Public Health ($n = 1$) from Frontiers; Applied Sciences ($n = 1$) and Technologies ($n = 1$) from MDPI; and Public Health Genomics ($n = 1$) from Karger.

The country column in Table 2 was populated with the affiliation of the first author’s institution. The UK ($n = 8$) was the most prolific, followed by Australia ($n = 3$), the US ($n = 2$), South Korea ($n = 2$), Norway ($n = 2$), Switzerland ($n = 1$), Germany ($n = 1$), Italy ($n = 1$), Malta ($n = 1$), and Malaysia ($n = 1$). European countries, including the UK, Norway, Switzerland, Germany, Italy, and Malta, comprised more than half of the total, demonstrating substantial academic effort in the European region.

#### Research themes

The analysis of the selected articles revealed three overarching themes: conceptual evolution, feasibility analysis, and technological advancement. Table 3 provides the annual number of publications for each of these three themes, offering a clear overview of the research trends related to DC.

**Table 3:** Research themes

Year	Conceptual evolution	Feasibility analysis	Technological advancement
2013	1	0	0
2014	0	0	0
2015	2	2	0
2016	0	1	0
2017	0	1	0
2018	1	0	0
2019	1	0	0
2020	2	2	2
2021	0	1	3
2022	0	1	2
Total	7	8	7

Conceptual Evolution: Articles pertaining to the theme of conceptual evolution explored the evolutionary history of the DC concept while emphasizing its ethical strengths. Early-stage publications highlighted the major debate surrounding broad informed consent compared to DC. These articles contended that the contemporary communication attributes of DC could effectively tackle apprehensions regarding broad informed consent with its superiority in autonomy, information, engagement, control, social robustness, and reciprocity [41]. Furthermore, these articles underscored the advantages of DC, especially its ethical merits in terms of transparency and efficiency, as well as its conformity with legal standards [32].

Feasibility Analysis: These articles examined the acceptances among various stakeholders in DC. Researchers employed a variety of methodologies to gather stakeholder opinions (Additional file 1). Several studies used quantitative methods, such as pilot tests and questionnaires, to assess individual preferences [42, 50]. Other researchers collected qualitative data, including perspectives on DC, attitudes towards digital-based interfaces, and intentions to use them, through focus group interviews [44, 45, 54]. There were also studies that used mixed methods, combining quantitative and qualitative data [34], or organized a multidisciplinary workshop with experts from various fields [35]. Participants in most studies showed positive feedback on digital-based consent; however, concerns were also raised regarding the risks of handling sensitive data and the necessity for improved identity verification procedures. Nonetheless, most participants expressed the belief that the benefits of DC outweighed its drawbacks.

Technological advancement: As the fundamental principle of DC necessitates digital interfaces, several articles have been dedicated to the implementation of DC systems. Significantly, there have been growing interests in incorporating blockchain technology into DC systems. Some researchers suggested blockchain-based consent management systems along with the use of smart contracts [48, 49, 53, 55, 57]. This scholarly attention has been directed towards leveraging the blockchain’s fundamental features, such as integrity, transparency, and accountability, to enhance the trustworthiness of digital-based consent management. However, since blockchain is not a security technology, concerns regarding privacy and security continued to be formidable obstacles.

#### Keywords & application areas

**Fig. 3:**
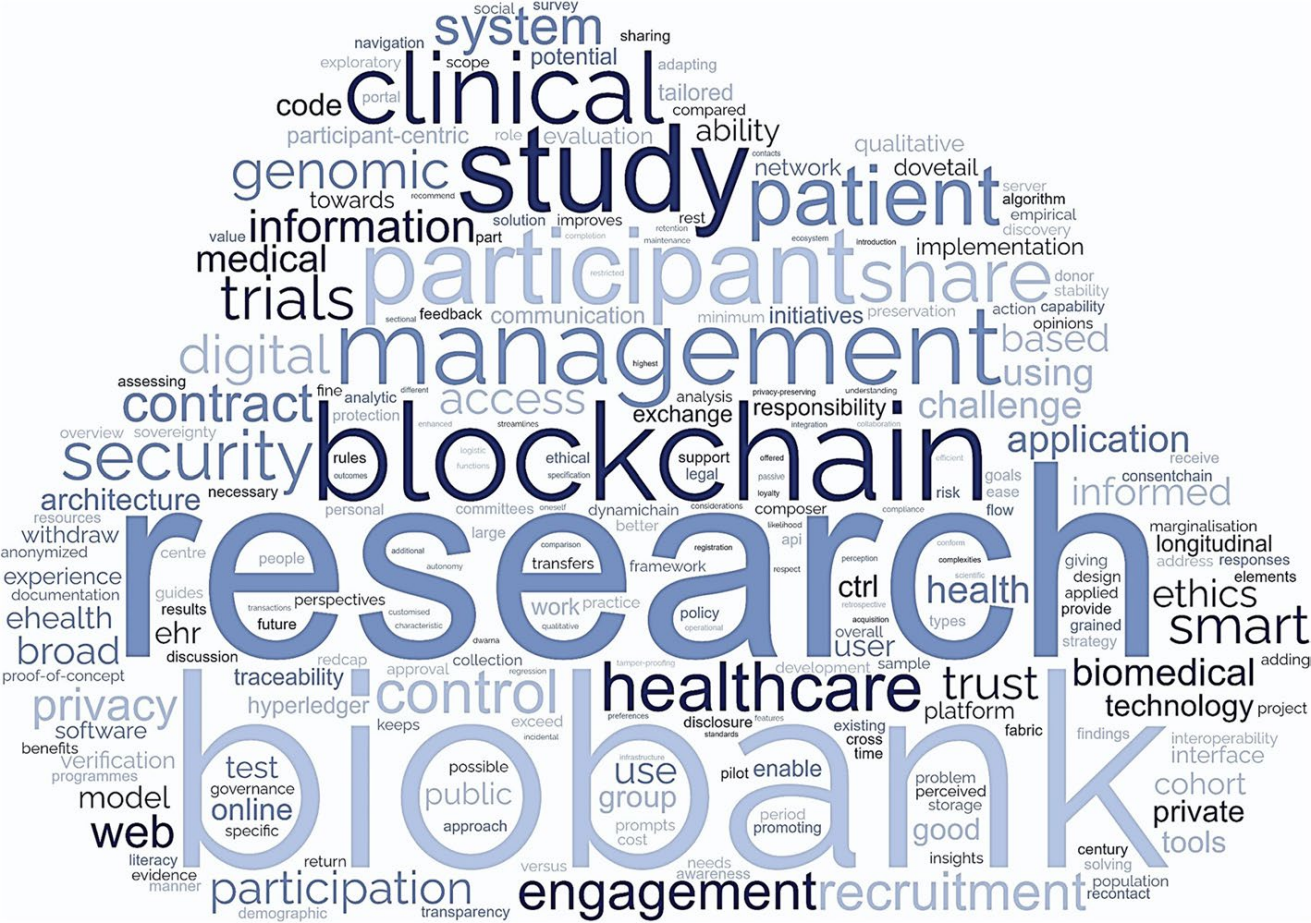
Keyword word cloud

Figure 3 presents the frequency of author-provided keywords as a word cloud. Since “dynamic,” “consent,” and “data” have natural occurrences in common, these were omitted to focus on other primary keywords. Table 4 displays the author-provided keywords categorized by related topics, including the omitted keywords from the word cloud.

**Table 4:** A summary of author-provided keywords

Consent methods	Application areas	Participant initiative	IT systems
$\bullet$ dynamic consent	$\bullet$ biobank	$\bullet$ participant-centric	$\bullet$ digital, online, web
$\bullet$ broad consent	$\bullet$ biomedical research	$\bullet$ engagement, participation	$\bullet$ software tools
$\bullet$ informed consent	$\bullet$ clinical trials, research	$\bullet$ research communication	$\bullet$ eHealth, EHRs, EPR
$\bullet$ formal consent model	$\bullet$ genomics	$\bullet$ ethics, equity	$\bullet$ blockchain, smart contract
$\bullet$ medical consent	$\bullet$ longitudinal cohort studies	$\bullet$ public trust	$\bullet$ digital consent
	$\bullet$ medical data	$\bullet$ autonomy, data sovereignty	$\bullet$ security, privacy
	$\bullet$ public health		$\bullet$ data protection

The key terms “research” and “biobank” were the most frequently recurring. These keywords, in addition to “clinical,” “trials,” and “study,” indicate the primary application areas for DC. DC is primarily used in biobanks; however, its application is expanding to other fields that utilize personal health data. As shown in Table 4, in addition to the biomedical and genomic fields, clinical trials and longitudinal cohort studies are emerging as new application areas for DC.

Additionally, the word cloud highlights keywords related to participant initiatives, including “participant,” “patient,” and “engagement.” These keywords reference data subjects’ sovereignty, which is a key concept in DC. Topics related to participant initiative include “patient-centered,” “engagement,” and “ethics.” The term “blockchain,” commonly used to implement DC systems, is also frequently mentioned. Information technology-related terms, such as “security” and “privacy,” have also emerged.

### Primary characteristics

#### Overview

The primary features of DC differentiate it from conventional consent methods. This section summarizes the findings from synthesizing the selected articles to identify its nature characteristics, focusing on the facilitators of and barriers to adopting DC in digital health ecosystems.

#### Facilitators

Flexibility: The ability to support a variety of consent mechanisms is suitable for the purpose and context of digital health ecosystems [32]. This flexibility of DC allows researchers to obtain consent in a more sophisticated manner than conventional consent methods [35]. DC is not a fixed method; rather, it allows for the selection of an approach that is appropriate for a range of situations. Conventional consent methods, such as broad informed consent, can also be used if needed; for example, individuals can consent to a wide range of data uses if they so desire. These various consent mechanisms are established depending on the type of researcher or specific data use.

Tailored options: DC provides tailored options regarding data utilization. Individuals could customize the degree of accessibility that consumers could be granted to personal health data according to the institution or group to which the consumers are affiliated [46]. Additionally, the method of communication with the DC system may vary from traditional paper-based forms to digital means, such as email, text message, or social networking sites, according to individual preferences [32]. Individuals can choose their preferred information, contact method, and frequency, and these can be changed at any time according to individual preferences.

Continuous two-way communication: Anywhere, individuals and data consumers are able to interact in real time, anywhere. Digital interfaces enable individuals to centrally manage their personal health data, including tracking consent history, thereby empowering them to take a more proactive stance in using their data [58]. Previously provided consents can be modified or withdrawn at any time in response to circumstances that are constantly evolving, courtesy of digital interfaces [43]. A previous study demonstrated that the digital-based consent system was highly preferred by individuals in comparison to the previous consent procedure [34].

Afterward information: Individuals can receive additional information that is derived from their data provision. The afterward information may include the most recent developments in research, primary findings, and clinical or analytical outcomes. It can be delivered in the preferred timeframe and fashion, with the degree of engagement customized to suit individual preferences. DC is an appropriate approach for reporting research progress, discoveries, and study outcomes [41]. Providing individuals with additional information heightens their awareness of how their data is being used. A previous study demonstrated that the individuals held a positive perception of digital interfaces for DC, especially with regard to feedback about research outcomes [45].

DC can meet internationally accepted ethical and legal standards. It is considered to enable transparent international co-research in situations where countries differ in the extent to which broad consent is authorized. The adoption of DC can help alleviate the ethical concerns associated with the use of broad consent and enable harmonized international research collaboration. Kaye Jane et al. argued that the DC model offers a flexible and responsive solution to deal with changing legal and ethical requirements due to the enhanced ease of participant recontact [32]. Most studies promoted DC solutions as an alternative to improve transparency and public trust in complex research networks [45].

#### Barriers

Excessive role to individuals: Individuals can be more engaged with DC by exercising granular control over the utilization of their health data, for instance. Some individuals may perceive DC as burdensome because of the excessive number of decisions it necessitates, which can lead to consent fatigue. If enormous amounts of information are provided to individuals to make fully informed decisions, there is a risk of making it difficult to distinguish between relevant and irrelevant information before providing consent [41]. In addition, any risk that DC may further promote consent fatigue would undermine the intentions of the approach [46]. It would also lead to an accountability problem in research for not ensuring mutual understanding.

Digital divide: The digital divide is an ethically challenging issue related to DC. Unlike other consent methods, DC requires users to interact with digital interfaces such as websites and mobile applications. These digital interfaces can be difficult for those who are unfamiliar with technologies, thereby alienating themselves from the process of utilizing personal health data prior to experiencing its benefits. Susan E. Wallace and José Miola were concerned that some populations, including the elderly or disadvantaged, may be unable or unwilling to participate in a technology-based consent process owing to a lack of interest, comprehension, or access to resources [54]. Given that older and disadvantaged people will suffer more from disease, it will be problematic under the principles of equality and justice. If individuals are not prepared to engage using the technology, there exists the potential to aggravate marginalization and disenfranchisement [46]. This may equally apply to communities, such as those in isolated areas of Australia, that lack access to technology or stable infrastructure, such as WiFi networks, to enable significant dependence on these tools. Ultimately, the digital divide may result in selection bias among the study population and lower the quality of research results.

System implementation: To benefit from DC, a system supporting its concepts should be implemented. The successful implementation of a DC system requires careful interface design and a robust architecture to establish trust with system users [45]. For instance, it is imperative that the DC system has the ability to interact with legacy systems that store personal health data. Furthermore, data consumers, such as researchers, require dedicated interfaces to furnish individuals with afterward information regarding their data uses. Notwithstanding recent technological advancements, implementing a DC system necessitates pragmatic investments of time, money, personnel, and the willingness of stakeholders. These additional burdens may have an adverse effect on the adoption of DC [46].

Privacy and security: There must be a secure connection between researchers and individuals for large amounts of traceable health information without risk of privacy exposure. Establishing a balance among security, privacy, accessibility, and usability will continue to be a difficult challenge [42]. Privacy and security are indispensable components of any information system, including DC systems [48]. Notably, individuals are more concerned about the disclosure of their information because health data contains sensitive personal information. Therefore, DC systems must be implemented alongside privacy and security technologies that data subjects are able to use with trust.

### Technical trends

The articles that examined technological advancements addressed system implementation and related technologies for applying the DC concept (Additional file 2). A discernible trend toward the implementation of DC systems utilizing blockchain technology has been identified. With two exceptions [52, 56] that put forth a web-based DC system, the majority of the articles examined the application of blockchain technology. Permissioned blockchain was mostly utilized, rather than permissionless public blockchain. The most widely used platform was Hyperledger Fabric [53, 55, 57]. Hyperledger Composer [48, 49] was also mentioned, but this platform has been deprecated since August 2019. One article used Hyperledger Besu [55]. Prominent digital assets that were stored on a blockchain network comprised consent histories and hash values pertaining to personal health data. The personal health data was stored separately in external storage rather than on the blockchain, and the hash value or identifier of the personal health data was stored on blockchain networks.

The design goals discussed in these studies were privacy, security, traceability, compatibility, and legal compliance. Since most health data contains sensitive information, most articles have considered privacy and security when implementing the DC system. Traceability is discussed with blockchain technology. Along with immutability, the representative characteristics of blockchain are accountability and transparency. All transactions that occur within a blockchain network are transparently recorded, including who initiated the transaction and when. These features enable individuals to track their data usage and follow their consent history within DC systems. However, compatibility and legal compliance were discussed less frequently than other design goals.

## Discussion

In digital health ecosystems, ethical dilemmas surrounding the use of personal health data have arisen. These ethical considerations derive from fundamental ethical principles, such as the Belmont Report and the Declaration of Helsinki. Regarding the ethical obligation of consent in digital health, respect for individuals, with an emphasis on their right to autonomy and self-determination, is fundamental to ethical medical research and practice. While conventional consent practices have been historically accepted, they have struggled in rapidly evolving digital health environments. DC is a more ethically preferable paradigm because it allows for continuous interaction with participants, thereby upholding individuals’ preferences.

The current research trends pertaining to DC within the health domain showed an increase in the annual number of related publications and citations, which indicates a growing interest in DC. Its primary application area was biobanks; however, several studies have discussed the scalability of the DC system. It has the potential to be used anywhere personal health data is used, from biomedical big data studies to multicenter clinical trials. As the types of collected personal health data diversify, DC’s potential application areas appear to be expanding. In summary, the growing academic focus on DC indicates a larger shift toward participant-centered research, which promotes the engagement of participants and respects their autonomy.

Within the digital health environment and the ethical landscape of consent, DC embodies adaptability regarding personal health data use. Its remarkable flexibility supports ethical principles of autonomy and justice, ensuring all individuals, regardless of their changing circumstances, have a voice in decisions about their data. Its platform for continuous two-way communication ensures that individuals are always informed and in control, thereby solving the data governance issues. Unlike previous static, one-time conventional consent practices, DC provides more possibilities for individuals to engage actively in the process of health data usage. By providing afterward information to individuals after consent, it helps their informed choice regarding data usage. Furthermore, DC offers alternative options to broad informed consent, which enables compliance with high ethical and legal standards. Ultimately, this not only reinforces the principles of respect for persons but also fosters the trustworthiness of research.

Although DC is a promising approach, its realization in practice presents ethical challenges. One representative issue is the digital divide, which raises justice and equity concerns. DC may inadvertently neglect passive participants, contradicting the principle of equal participation [41]. Since DC relies on digital interface proficiency, it may exclude vulnerable populations, such as the elderly, disadvantaged, and remote residents [45, 59]. This concern may stem from the misconception that DC necessitates active participation. However, DC allows a variety of approaches to accommodate various circumstances, so passive individuals can still use broad-informed consent for a more inclusive approach. Another challenge was reconciling the concept with real-world environments, especially complying with ethical and legal standards including HIPAA and GDPR. DC requires sharing and collaborating on personal health data while meeting ethical and legal requirements in multiple jurisdictions. Therefore, it is necessary to explore not only technological advancement but also ethical suitability for implementing the concept of DC.

Moreover, there are still several obstacles to implementing DC systems. Privacy and security concerns are the most significant obstacles to the implementation of DC. This indicates the need for privacy-enhancing techniques such as data encryption, authorization, and authentication [48, 53, 55]. Future research should also address external factors such as compatibility and legal compliance, in addition to privacy and security concerns. Compatibility refers to the capability of the DC system to integrate seamlessly with legacy systems, such as hospital information systems that store personal health data and biobank systems. Numerous stakeholders considered compatibility and interoperability as vital factors to utilize personal health data [49, 52, 59, 60]. Further, blockchain technology faces potential conflicts with the ethical principle of the General Data Protection Regulation (GDPR), the right to be forgotten, as it struggles with data deletion [61]. Compliance with major regulations such as GDPR and the Health Insurance Portability and Accountability Act (HIPAA) is not only a legal requirement but also an ethical obligation to protect the rights of participants [51, 62].

This study has the limitation that it does not provide an in-depth discussion of related technical research advancements and ethical requirements, as its primary objective is to identify facilitators of and barriers to adopting DC in digital health environments. Nevertheless, having summarized research trends on DC and pointed out research gaps that need to be filled, the present study demonstrates directions for future research. Based upon the review, we summarized that DC has strengths in several ethical problems, including respect for autonomy and trustworthiness. However, so far, it still raises justice issues regarding the digital divide, the cost of implementation, and accountability. We suggest these concerns should be re-addressed by widening the concept of DC as a tailored adaptation of consent practice in various circumstances. Furthermore, it is necessary to explore the possibility of DC in various ethical-legal settings of digital health research. Without breakthrough technological advancements to solve privacy and security issues, otherwise, DC may remain a difficult concept to implement in a real-world research environment, despite its possibilities.

## Conclusion

This study conducted a scoping review on DC to identify barriers to and facilitators of its adoption in digital health environments. Traditional consent methods are predominantly one-time and static, rendering them unsuitable for digital health environments. The findings revealed several characteristics of DC that are suitable for the digital health environment owing to its flexible and dynamic nature. Furthermore, this study discussed challenging issues associated with the implementation of a DC system, as determined by a synthesis of limitations, recommendations, and knowledge gaps mentioned in prior literature. Future research should emphasize privacy and security, compatibility with legacy systems, and legal compliance to establish a technical framework that suits the diverse needs of various DC stakeholders.

### Supplementary information

**Additional file 1.**

**Additional file 2.**

## Data Availability

All data generated or analysed during this study are included in this published article.

## Abbreviations

DC: Dynamic Consent
FHIR: Fast Healthcare Interoperability Resources
HIPAA: Health Insurance Portability and Accountability Act
GDPR: General Data Protection Regulation
