# Supplementary material for: Identifying facilitators of and barriers to the adoption of dynamic consent in digital health ecosystems: a scoping review
Source: BMC Med Ethics. 2023 Dec 1;24:107. doi: 10.1186/s12910-023-00988-9 (PMC10693132; doi:10.1186/s12910-023-00988-9)
Supplement: Supplementary file 1 — Additional file 1. [file 12910_2023_988_MOESM1_ESM.pdf]

**Additional file 1.** A summary of articles on feasibility analysis

| Author, Year of publication | Study design | Purpose | Participants | Principal findings | Limitations |
|---|---|---|---|---|---|
| Thiel, D.B. et al., 2015 [1] | Pilot test | to evaluate user experiences with an online portal for DC in biobank research | a total of 187 pilot testers (Michigan citizens and University of Michigan students) | ● over 50% of participants were willing to use and recommend the tested portal if it went live<br>● concerns were raised about the identity verification procedure | not representative of the general population since the pilot testing sample was a convenience sample |
| Tears, H.J. et al., 2015 [2] | Focus group interview | to investigate the viewpoints of biobank participants regarding the web-based DC interface | a total of 32 biobank participants (three biobanks governed by NHS Hospital Trust | ● positive to track consent history, receive additional information about research progress, and specify the frequency and method of receiving additional information | a preliminary local study with a small sample size, and demographic considerations were not considered when selecting participants |
| Spencer, K. er al., 2016 [3] | Focus group interview | to assess patient impressions of the continuous communication between data subjects and consumers in a DC system | a total of 40 participants (35 patients with rheumatic disease and 5 from a public involvement health research network) | ● overall, 98% of participants believed that the benefits of utilizing personal health data with dynamic consent outweighed the drawbacks<br>● concerns about the negative repercussions of sensitive data falling into the wrong hands | undertaken on a select population of individuals with chronic rheumatic disease and with little consideration for generalizability |
| Budin-Ljøsne et al., 2017 [4] | Interdisciplinary workshop | to explore whether DC can aid participant recruitment and contact maintenance | Diverse DC stakeholders (organized by the University of Oxford & COST Action CHIP ME) | ● DC brings pragmatic solutions to consent management issues<br>● a competent workforce is required for the implementation and maintenance of a DC system | not mentioned |
| Despotou, G. et al., 2020 [5] | Focus group interview & questionnaire | to assess how patients perceive blockchain-based digital consent applications in the context of diabetes care | a total of 36 participants (23 patients with diabetes and 13 staff in the practice) | ● lacked awareness of existing consent processes and could not recall previously providing consent<br>● received positive feedback, with patients acknowledging the utility of the digital consent application | participants were recruited from a local general practitioner's practice |

| | | | | | |
|---|---|---|---|---|---|
| Pacyna, J.E. et al., 2020 [6] | Survey | to evaluate the stability of the donor preferences specified during biobank enrollment | a total of 1,164 participants (Mayo Clinic biobank) | ● overall, 94% of participants initially restricted sample availability when they enrolled in the biobank, but they were later comfortable with wider sample availability<br>● participants need to be able to contact the biobank at any time to update their preferences regarding the use of their donated human-derived materials | inappropriate to generalize results to other populations or research settings |
| Wallace, S.E., and Miloa, J., 2021 [7] | Focus group interview | to investigate the opinions of existing study participants regarding the addition of a DC interface | four focus groups, each comprising 3-6 people (EXCEED longitudinal cohort study) | ● positive regarding the ability to update and revoke consent | few participants with a favorable bias toward the study |

DC=Dynamic consent

**References**


1. Thiel, D.B., Platt, J., Platt, T., King, S.B., Fisher, N., Shelton, R., Kardia, S.L.: Testing an online, dynamic consent portal for large population biobank research. Public Health Genomics 18(1), 26–39 (2015)
2. Teare, H.J., Morrison, M., Whitley, E.A., Kaye, J.: Towards ‘engagement 2.0’: Insights from a study of dynamic consent with biobank participants. Digital Health 1, 2055207615605644 (2015)
3. Spencer, K., Sanders, C., Whitley, E.A., Lund, D., Kaye, J., Dixon, W.G.: Patient perspectives on sharing anonymized personal health data using a digital system for dynamic consent and research feedback: a qualitative study. Journal of medical Internet research 18(4), 66 (2016)
4. Budin-Ljøsne, I., Teare, H.J., Kaye, J., Beck, S., Bentzen, H.B., Caenazzo, L., Collett, C., D’Abramo, F., Felzmann, H., Finlay, T., et al.: Dynamic consent: a potential solution to some of the challenges of modern biomedical research. BMC medical ethics 18(1), 1–10 (2017)
5. Despotou, G., Evans, J., Nash, W., Eavis, A., Robbins, T., Arvanitis, T.N.: Evaluation of patient perception towards dynamic health data sharing using blockchain based digital consent with the dovetail digital consent application: A cross sectional exploratory study. Digital Health 6, 2055207620924949 (2020)
6. Pacyna, J.E., McCormick, J.B., Olson, J.E., Winkler, E.M., Bublitz, J.T., Hathcock, M.A., Sharp, R.R.: Assessing the stability of biobank donor preferences regarding sample use: evidence supporting the value of dynamic consent. European Journal of Human Genetics 28(9), 1168–1177 (2020)
7. Wallace, S.E., Miola, J.: Adding dynamic consent to a longitudinal cohort study: A qualitative study of exceed participant perspectives. BMC Medical Ethics 22(1), 1–10 (2021)
