# Supplementary material for: Identifying facilitators of and barriers to the adoption of dynamic consent in digital health ecosystems: a scoping review
Source: BMC Med Ethics. 2023 Dec 1;24:107. doi: 10.1186/s12910-023-00988-9 (PMC10693132; doi:10.1186/s12910-023-00988-9)
Supplement: Supplementary file 2 — Additional file 2. [file 12910_2023_988_MOESM2_ESM.pdf]

Additional file 2. The characteristics of articles on technological advancement

Author, Year of publication	Blockchain platform	Digital assets	Design goals				
			**security**	**privacy**	**traceability**	**compatibility**	**legal compliance**
Mamo, N. et al., (2020)	Hyperledger Composer	consent	X	X	X		X
Albanese, G. et al., (2020)	Hyperledger Composer	contract, trial	X	X	X	X	
Haas, M.A. et al., (2021)	-	-	X	X	X	X	
Kim, T.M. et al., (2021)	Hyperledger Fabric	consent, data hash	X	X	X		
Albalwy, F. et al., (2021)	Hyperledger Besu	consent, profile	X	X	X		
Appenzeller, A. et al., (2022)	-	-	X	X		X	X
Huh, K.Y. et al., (2022)	Hyperledger Fabric	consent, data hash	X	X	X		

References

1. Mamo, N., Martin, G.M., Desira, M., Ellul, B., Ebejer, J.-P.: Dwarna: a blockchain solution for dynamic consent in biobanking. European Journal of Human Genetics 28(5), 609–626 (2020)
2. Albanese, G., Calbimonte, J.-P., Schumacher, M., Calvaresi, D.: Dynamic consent management for clinical trials via private blockchain technology. Journal of ambient intelligence and humanized computing, 1–18 (2020)
3. Haas, M.A., Teare, H., Prictor, M., Ceregra, G., Vidgen, M.E., Bunker, D., Kaye, J., Boughtwood, T.: 'ctrl': an online, dynamic consent and participant engagement platform working towards solving the complexities of consent in genomic research. European Journal of Human Genetics, 1–12 (2021)
4. Kim, T.M., Lee, S.-J., Chang, D.-J., Koo, J., Kim, T., Yoon, K.-H., Choi, I.-Y.: Dynamichain: Development of medical blockchain ecosystem based on dynamic consent system. Applied Sciences 11(4), 1612 (2021)
5. Albalwy, F., Brass, A., Davies, A., et al.: A blockchain-based dynamic consent architecture to support clinical genomic data sharing (consentchain): Proof-of-concept study. JMIR medical informatics 9(11), 27816 (2021)
6. Appenzeller, A., Hornung, M., Kadow, T., Krempel, E., Beyerer, J.: Sovereign digital consent through privacy impact quantification and dynamic consent. Technologies 10(1), 35 (2022)
7. Huh, K.Y., Jeong, S.-u., Moon, S.J., Kim, M.-J., Yang, W., Jeong, M., Song, I., Kwak, Y.-G., Lee, S., Kim, M.-G.: Metory: Development of a demand-driven blockchain-based dynamic consent platform tailored for clinical trials. Insights in Regulatory Science: 2021 (2022)
